# Strategies for Regulating the Release Kinetics of Bioactive Compounds from Biopolymeric Hydrogels

**DOI:** 10.3390/gels11120986

**Published:** 2025-12-08

**Authors:** Mizanur Rahman, Shahla Teimouri, Poly Rani Roy, António Raposo, Hmidan A. Alturki, Stefan Kasapis

**Affiliations:** 1 School of Science, RMIT University, Bundoora West Campus, Plenty Road, Melbourne, VIC 3083, Australia; s3985616@student.rmit.edu.au; 2 Lactalis Australia, 842 Wellington Rd, Rowville, VIC 3178, Australia; shahlateimouri@gmail.com; 3 Department of Chemistry, Jagannath University, Dhaka 1100, Bangladesh; polyrani92@gmail.com; 4 CBIOS (Research Center for Biosciences and Health Technologies), ECTS (School of Health Sciences and Technologies), Lusófona University, Campo Grande 376, 1749-024 Lisboa, Portugal; 5 King Abdulaziz City for Science & Technology, Wellness and Preventive Medicine Institute—Health Sector, Riyadh 11442, Saudi Arabia; halturki@kacst.edu.sa

**Keywords:** controlled release, diffusion coefficient, hydrogels, natural bioactives, polymer crosslinking, release media

## Abstract

Bioactive compounds are widely recognized for their ability to enhance health and prevent diseases due to their various biological activities. However, these compounds are very sensitive to environmental factors, which can reduce their solubility, bioavailability, permeability, and stability, necessitating carriers to protect and ensure targeted delivery. To develop an effective delivery system, it is essential to assess the key factors that influence the release behaviour of bioactive compounds. Therefore, the primary aim of this study is to evaluate how the conditions of the release media, the attributes of hydrogels, and the characteristics of the entrapped bioactive compounds regulate the release kinetics of these compounds. Prior to create suitable carriers, it is essential to comprehend the mechanisms of digestion and absorption of these compounds. Consequently, absorption and the factors influencing stability and bioavailability of bioactives were reviewed first. The conditions of release media, especially the pH, ionic characteristics, temperature, and the nature of solvent served as a critical determinant in the release of bioactive substances by affecting the functional groups, electrostatic interactions between carrier and entrapped bioactive compound, dissociation and conformational changes in polymers. The properties of delivery systems can be controlled using polymers, crosslinkers, plasticizers, and specific environmental factors. The application of dual crosslinkers or a combination of physical and chemical crosslinkers enhanced the efficiency of the crosslinking process, subsequently improving the overall release profile of bioactive compounds from the matrices. Therefore, this review explored several options for enhancing the delivery system.

## 1. Introduction

Currently, researchers are increasingly focused on bioactive compounds that originate from seeds, food sources, and products resulting from fermentation processes [1]. These compounds play a vital role in metabolic processes and can exert positive effects on human health, including antioxidant properties, modulation of enzyme activities, regulation of gene expression, and interaction with receptors, among various other functions [2]. The most notable bioactive compounds pertinent to the human diet include carotenoids, phenolic compounds, glucosinolates, vitamins, tocopherols, phytosterols, organosulfur compounds, flavonoids, fatty acids, dietary fibre, and folates, among others [3]. An effective strategy for creating novel functional foods involves the integration of peptides, probiotics, vitamins, antioxidants, and various other natural bioactives into food formulations [4,5]. However, the poor solubility, instability, and low bioavailability of these bioactive compounds have considerably restricted their application within the food industry [6].

Utilizing suitable delivery systems to entrap the compounds can alleviate these challenges to a degree [7]. Encapsulation is a technique for safeguarding and delivering bioactive compounds to their intended site by embedding these active ingredients within the structures of wall materials [8]. The food industry anticipates a growing demand for intricate characteristics in food ingredients, including delayed release, stability, thermal protection, and an appropriate sensory profile. Achieving these properties frequently necessitates the use of a delivery system [9]. Food encapsulation possesses numerous intriguing and distinctive characteristics; however, its application remains difficult, mainly because of various side effects, including toxicity, lack of specificity, low bioavailability, short duration of drug delivery, and swift degradation [10]. It is essential to use materials that are generally recognised as safe (GRAS) [11].

**Figure 1:**
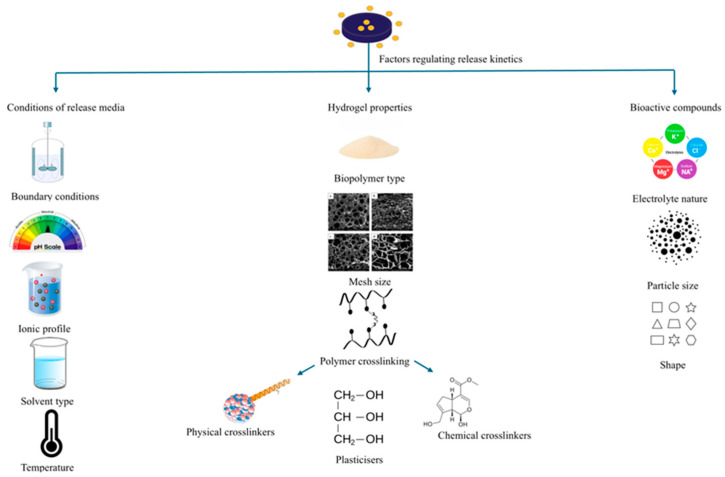
Strategies employed to regulate the release rate from biopolymeric hydrogels.

Recently, high concentration of biopolymers has been considered more advantageous for encapsulation, as it results in a more compact and coherent gel matrix that enhances the barrier effect [12]. With their unique eco-friendly and adaptable properties, biopolymers play a vital role in developing sustainable and innovative formulations [13]. The presence of hydroxyl, amino, or carboxyl groups in natural biopolymers increases their chemical reactivity and versatility, making them comparable to synthetic polymers [14,15]. Furthermore, a significant advantage of utilizing biopolymers is their capacity to be produced from the non-edible components of plants or animals. The primary categories of biopolymers that can be derived from biomass waste, along with examples of their respective sources are presented in Figure 1, adapted from [16].

Controlled release systems provide sophisticated platforms for creating novel formulations and dietary approaches, allowing precise regulation of the release and activity of bioactive food components [17]. Controlled-release delivery systems can safeguard bioactives from hostile conditions, including light, pH levels, temperature fluctuations, and processing environments, thereby enhancing their bioavailability [18]. Additionally, controlled-release delivery systems have garnered interest in active food packaging, leading to an enhanced shelf life of food items [19]. As a controlled delivery system, hydrogels have attracted significant attention and have applications in various areas, including synthetic tendons [20], contact lenses [21], scaffolds for tissue engineering [22], environmental remediation [23], and drug delivery [24], as well as in agriculture and biosensors [25]. Hydrogels are 3D networks of polymers that absorb water or biological liquid while maintaining their structural integrity [26]. The properties of hydrogels, including their physicochemical, mechanical, and biocompatible characteristics, are influenced by the type of polymer used, the ratio of its constituents, the overall composition, and the methods employed in their fabrication [27].

As interest in controlled release delivery systems continues to rise within the food and pharmaceutical sectors, numerous efforts are being undertaken to assess the factors influencing the release characteristics of natural bioactive compounds from these delivery systems. Bioactives can be released from hydrogels through diffusion-controlled, swelling-controlled, or chemically controlled mechanisms [28]. The primary mechanism for the transfer of these ingredients is typically the process of diffusion [29]. It is common to study release kinetics using various mathematical models, the most widely applied being zero-order, first-order, Higuchi, Korsmeyer–Peppas, and Hixson–Crowell models [30]. When designing a delivery system for the micronutrients, it is essential to consider their physicochemical properties, the types and concentrations of additives (such as plasticizers, and crosslinkers) incorporated in the carriers, and environmental factors like pH, temperature, and ionic profile [31]. The effect of the release medium on drug release from biopolymeric matrices was investigated, revealing that varying drug release profiles were influenced by the medium’s pH, osmolarity, and temperature [32]. Another study investigated the impact of various crosslinkers on the release of bioactive compounds. Swelling and in vitro tests indicated that the release was significantly influenced by time, crosslinker type and concentration, and medium pH [33]. Crosslinkers, in fact, are regarded as effective tools for achieving sustained release by enhancing the structural characteristics of biopolymers [34]. A review showed that bioactive compounds, depending on their characteristics (concentration, structure, and reactive groups), modified the film structure and permeability by acting as either plasticizers, anti-plasticizers or crosslinkers [35].

Considering these key regulatory factors, this communication provides a comprehensive overview of the strategies utilized to regulate the release behaviour of bioactive molecules from biopolymeric matrices. This includes the selection of release media conditions, such as pH and temperature, various physical and chemical crosslinking treatments, the incorporation of additives, such as plasticizers, and the inherent characteristics of the bioactive compounds. Finally, we discuss the potential for predicting and regulating the release rate of bioactive components from biopolymeric hydrogels.

## 2. Absorption of Bioactive Compounds

Understanding the digestion and absorption mechanisms of different bioactive ingredients is essential prior to developing appropriate carriers for these substances. The gastrointestinal (GI) tract is the primary site for digesting and absorbing food components. In the stomach, food is mechanically broken down into a thick, semifluid mixture called chyme, which then moves into the duodenum for further processing. Some chemical digestion also occurs in the stomach, breaking down macromolecules such as proteins, fats, and polysaccharides. Once in the small intestine, these nutrients are absorbed into the body [6]. The physiological functions and absorption mechanisms of different bioactive compounds are outlined in Table 1.

Vitamin D is mainly absorbed via passive diffusion as well as through a mechanism that includes membrane transporters, particularly cholesterol carriers [42]. Bioactive polypeptides, bioactive polysaccharides and unsaturated fatty acids are taken up through carrier-mediated transport, with the carriers being uniformly distributed throughout various regions of the small intestine [45,50,55]. Furthermore, the receptor-mediated transport mechanism plays a crucial role in the absorption of bioactive polypeptides [56]. Carrier-mediated diffusion primarily facilitates the absorption of polyphenols, including tea catechins [57]. A significant portion of polyphenols and certain larger molecules are not absorbed in the small intestine; consequently, these compounds arrive in the large intestine, where they undergo metabolism by the microbiota into smaller molecules [58]. Hydrophilic substances, including polyphenols and most of the drugs, exhibit a more straightforward absorption mechanism compared to lipids. Hydrophobic components are primarily absorbed through passive diffusion and receptor-mediated transport. For example, resveratrol may be taken up by passive diffusion or by interacting with membrane transporters such as integrins [52]. Additionally, the low aqueous solubility of hydrophobic compounds further leads to low absorption rate; thus, enhancing the aqueous solubility of these hydrophobic ingredients through the use of carriers is crucial [6].

## 3. Factors Influencing Stability and Bioavailability of Natural Bioactive Absorption of Bioactive Compounds

The low absorption efficiency of food bioactives leads to decreased bioavailability, as these compounds need to pass through gastrointestinal barriers before being taken up by the body. Moreover, their stability can be affected by multiple environmental factors. In this regard, the main factors influencing the absorption of bioactive ingredients are summarized in Table 1.

The stability of bioactive compounds is strongly influenced by environmental factors such as extreme pH, light exposure, heat, and oxygen. For example, the antioxidant activity of carotenoids largely depends on their conjugated double bonds, which are highly susceptible to oxidation [59]. Additionally, interactions with other food components, such as proteins and fats, can lessen their effectiveness [60]. The limited solubility of curcumin, a naturally occurring polyphenolic substance derived from turmeric, in water and its swift degradation at physiological pH levels lead to reduced bioavailability and suboptimal pharmacokinetics. Consequently, only a minor fraction of curcumin consumed orally is effectively digested and absorbed by the human body [61].

The harsh conditions of the gastrointestinal (GI) tract represent the second factor affecting bioavailability. Accurate knowledge of how food components are broken down in the stomach is essential for evaluating the bioaccessibility of phytochemicals. This stage of digestion involves both mechanical processes and the action of gastric secretions. Gastric juice is composed of hydrochloric acid, pepsinogens, lipase, mucus, electrolytes, and water. Under fasting conditions, the secretion rate of gastric juice ranges from about 1 to 4 mL/min, increasing to 1–10 mL/min following food intake. Hydrochloric acid contributes to protein denaturation and activates pepsin, while peristaltic waves help reduce the size of solid food particles to approximately 1–2 mm. In healthy individuals, fasting gastric pH typically ranges from 1.3 to 2.5, but it generally rises above 4.5 after a meal, depending on the food’s buffering capacity [62]. For instance, studies indicate that flavonoids and hydroxycinnamoyl acid experienced degradation rates of 84% and 80%, respectively, when subjected to in vitro GI environments, while vitamin C is reported to be degraded by 91% during intestinal digestion state [63]. Furthermore, digestive enzymes such as pepsin in the stomach and trypsin in the intestine can break down bioactive proteins and peptides, leading to a reduction in their activity. For example, quercetin is a well-known plant flavonoid that exhibits a variety of pharmacological effects. Nevertheless, its use in the pharmaceutical industry is constrained by several factors, including inadequate solubility, low bioavailability, limited permeability, and instability within the gastrointestinal tract [64]. Most importantly, prior to the bioactive components executing their intended functions, they might have already been degraded or digested. For this reason, the digestion occurring within the gastrointestinal tract is a great challenge that must be addressed to enhance their absorption.

Another barrier to absorption is the mucus layer that envelops the entire surface of the gastrointestinal tract. This mucus layer is mainly made up of glycoproteins, lipids, and discarded cellular materials [65]. Mucus is a semipermeable barrier that facilitates the exchange of various nutrients while simultaneously obstructing the entry of most bacteria and pathogens to the surfaces of epithelial cells [66]. The slow diffusion of food components through the mucus can restrict their access to enterocytes for absorption. Hydrophobic bioactive compounds, in particular, may interact with mucin proteins, which decreases their permeability through the mucus layer. The molecular structure of micronutrients significantly influences their absorption [67]. The results indicated that the bioaccessibility of phenolic compounds differed depending on their structures, with a clear relationship between molecular conformation and stability. For instance, it is well recognized that compounds with higher molecular weight, such as oligomeric proanthocyanidins and complex lipids, cannot permeate intestinal cells unless they undergo prior degradation [68]. Furthermore, the sugar component of flavonoids has been identified as an essential factor in their absorption in humans. When flavonoids are joined to an extra rhamnose unit, as seen with quercetin derived from tea, they must reach the large intestine to allow the intestinal microbiota to cleave the sugar moieties before absorption happens [69]. Still, it is not solely the chemical structure of bioactive food compounds that impacts their absorption; their isomeric configuration also plays a considerable role [6]. Like certain pharmaceuticals, flavonoids with varying stereochemistry show differing levels of bioavailability. This is exemplified by the bioavailability of (−)-epicatechin compared to (+)-catechin [70], and the superiority of Z-isomerization over all other isomers of carotenoids [71].

The various transport mechanisms, such as active transport, passive and facilitated diffusion, occurring within the intestinal lumen are also critical determinants of the bioavailability of consumed foods and drugs [58]. In summary, it is essential to develop strategies that overcome these challenges and improve the bioavailability of bioactive compounds. Each class of bioactives possesses unique chemical characteristics (hydrophilic or lipophilic), biological functions, and varying degrees of sensitivity. The limited solubility and susceptibility to oxidation of several vitamins, including vitamins A, D, E, and K, present significant obstacles to their application. Techniques such as nano-encapsulation and micro or nano-emulsion, utilizing nanoscale dimensions, can greatly enhance their availability and stability [72]. Polyunsaturated fatty acids, such as DHA and EPA, are especially prone to oxidation. Encapsulation techniques can effectively protect them from degradation [73]. Many bioactive proteins and peptides are prone to denaturation and exhibit low absorption in the gastrointestinal tract; however, encapsulation within nanoparticles or microparticles can protect these compounds and enable their targeted delivery to the intestine [74].

Numerous criteria must be met in the preparation of carriers as well. It is crucial that delivery carriers remain stable and prevent premature release of encapsulated compounds in the stomach’s acidic environment. Furthermore, carriers should be able to diffuse proficiently through the intestinal mucus layer to reach the surface of enterocytes rapidly, rather than being removed by the renewal of mucus. Particles that are hydrophilic and charge-neutral are more likely to pass the mucus layer with ease [75].

## 4. Entrapment of Bioactive Compounds

### 4.1. Polymers Used for Entrapment of Bioactive Compounds

A biopolymer is a type of polymer that originates from natural sources and is synthesized by living organisms. These polymers generally consist of repeating structural units, called monomers, which are linked together through covalent bonds. Biopolymers are defined as polymers obtained from renewable resources, including both biological and fossil-based biodegradable polymers [76]. Numerous polymers are employed as carrier materials for bioactive compounds. These include polylactic acid, polyhydroxyalkanoates, gelatin, β-glucans, alginate, dextran, starch, cellulose, chitosan, chitin, pectin, gums, collagen, zein, and hyaluronic acid, among various others [77].

### 4.2. Fabrication of Biopolymer Matrices

Hydrogels are widely utilized in biotechnology, including drug delivery, tissue engineering, food applications, cosmetics, and environmental remediation, requiring fabrication processes that ensure appropriate physico-mechanical properties. The properties of hydrogels are affected by factors such as the source of the biopolymer, its concentration or feed ratio, and the specific method used for hydrogel preparation. One common approach involves dissolving a single biopolymer in a solvent to form a three-dimensional hydrogel network. Another prevalent strategy is the creation of composite hydrogels, consisting of two or more polymers crosslinked through either single-step or multi-step processes. The presence of polar functional groups within the polymer chains, such as –NH_2_, –COOH, –OH, –CONH_2_, –CONH, and –SO_3_H, enhances the hydrophilic nature of the network and promotes water uptake [78]. The three-dimensional architecture of hydrogels is essential for maintaining their structural integrity and water absorption capacity [79].

#### 4.2.1. Single-Polymer Hydrogel

A single-polymer hydrogel consists of a network formed from only one type of monomer, serving as the basic structural unit of any polymer network. This type of polymer can be formed with [80,81,82,83,84] or without [85,86,87,88,89,90,91] addition of any crosslinking agent. Owing to their unique features—including high molecular weight, porous 3D architecture, hydrophilicity, and biocompatibility—many natural polysaccharides, such as starch, cellulose, chitosan, agarose, and alginate, as well as proteins like collagen and gelatin, have been employed in hydrogel fabrication. However, in most instances, the effectiveness of this category of biopolymer hydrogels becomes limited when no crosslinker, such as genipin, is incorporated. Uncrosslinked biopolymer solutions often fail to form stable gels, leading to rapid dissolution and burst release, thereby necessitating crosslinking to achieve controlled release.

#### 4.2.2. Multi-Polymer Hydrogel

Multi-polymer hydrogels are composed of two or more polymers, with at least one exhibiting hydrophilic properties. These hydrogels are typically fabricated using crosslinkers and/or fillers to improve their physico-mechanical characteristics. Composite hydrogels can be prepared via physical interactions, such as hydrogen bonding and hydrophobic forces, or through chemical methods like covalent crosslinking. A study indicates that hydrogels made from 1.5% genipin-crosslinked gelatin and chitosan demonstrate significant potential for on-demand drug delivery applications, particularly in the context of osteoarthritis treatment [92]. Xanthan gum–starch composite polymer has also been identified as a promising polysaccharide-based hydrogel, suitable for use as a drug delivery system [93]. Multi-polymer hydrogels also encompass interpenetrating polymer networks (IPNs), formed when a secondary hydrogel network is polymerized within an already polymerized hydrogel. This is typically achieved by immersing the pre-formed hydrogel in a solution containing monomers and a polymerization initiator. IPNs can be classified into two types: a full IPN, produced in the presence of a crosslinker, and a semi-IPN, formed without a crosslinking agent, where linear polymers are embedded within the original hydrogel network [94]. An example of a semi-IPN is the incorporation of linear cationic polyallylammonium chloride into acrylamide/acrylic acid multi-polymer hydrogels. This configuration not only enhanced the mechanical strength but also enabled a completely reversible pH-responsive release of theophylline [95]. The primary benefits of interpenetrating networks (IPNs) are the ability to make relatively dense hydrogel matrices that exhibit enhanced stiffness and toughness in their mechanical properties, along with the capacity to control their physical properties. Drug loading is usually done simultaneously with the polymerization of the interpenetrating hydrogel phase [96].

Polymer network systems (IPNs) consisting of carrageenan and guar gum, loaded with metronidazole as the model drug, were synthesized utilizing the microwave irradiation technique. The results indicated that the samples exhibited thermal stability and possessed a lower critical solution temperature ranging from 30 to 60 °C. Based on this investigation, it can be concluded that these IPNs are suitable for use as controlled release drug delivery systems, demonstrating potential for targeted drug delivery due to their responsiveness to external stimuli [97]. In another study, a pH-responsive interpenetrating polymer network was developed by combining two polysaccharides, Moringa bark gum (MOG) and carrageenan (CRG), using microwave irradiation, with metronidazole (MTZ) included as a model drug. The matrices possessed all the essential attributes necessary for an effective controlled delivery system, in addition to functioning as a tissue scaffold [98]. Successful in vitro trials have been conducted; however, extensive exploration is required, particularly in various animal models and through clinical trials, prior to application.

##### Hybrid Crystalline–Hydrogel Composites

While the multipolymer hydrogels discussed above combine two or more polymers to tune release properties, another emerging strategy involves hybrid crystalline–hydrogel composites. In these systems, crystalline drug domains are embedded within a hydrogel—often a single polymer—to achieve sustained and highly controlled release. Long-term stability studies at 4 °C demonstrated that crystalline amylase exhibits greater stability and prolonged release compared to its amorphous counterpart [99]. Amorphous systems, in contrast, frequently compromise protein stability due to exposure to stressors such as aqueous–organic interfaces, hydrophobic surfaces, detergents, elevated temperatures, and vigorous agitation. For instance, mechanical agitation of insulin can induce aggregation into amyloid-type fibrils, reducing its efficacy and bioavailability, while the gene therapy Zolgensma has a short 14-day shelf life, cannot be agitated, and must be stored at 2–8 °C [100]. These limitations restrict wider application. To address this, hybrid crystalline–hydrogel composites have been developed that stabilize proteins against thermal denaturation even at 50 °C and allow excipient-free protein delivery via mechanical release from a syringe [100]. Crystallization of insulin within agarose and fluorenylmethoxycarbonyl-dialanine (Fmoc-AA) hydrogels has also been successfully achieved, with insulin crystals remaining stable at 50 °C for seven days. Furthermore, insulin crystals grown in Fmoc-AA hydrogels exhibited enhanced thermal stability, remaining intact up to 60 °C over 24 h [101]. These findings highlight that the choice of hydrogel matrix significantly influences the physicochemical properties of the crystals, suggesting that crystallization within different hydrogel systems offers a promising approach to expanding the range of novel biopharmaceutical formulations.

## 5. Key Factors Regulating the Release Rate of Bioactive Compounds

The primary factors influencing controlled release include the characteristics of bioactive components, the properties of the matrix, and the conditions of the surrounding release media, as illustrated in Figure 1. Accordingly, the upcoming sections will explore these three major strategies, supported by a review of recent studies.

### 5.1. Conditions of Release Media

The environmental conditions play a significant role in determining the appropriate structure necessary for regulating the release of the bioactive compound. This is because the conditions of the release media are crucial in influencing the structure of the hydrogels, which in turn impacts the release rate of the entrapped nutrients. Some examples are listed in Table 2.

#### 5.1.1. Boundary Conditions

Boundary conditions, either stationary or moving, play a vital role in controlling the mass flux of diffusants from the hydrogels. Purely molecular diffusion due to a concentration gradient in stationary conditions can be easily accelerated providing moving boundary conditions in the system where the release of the bioactives is the combined effect of the concentration gradient and the motion of the release medium. In stationary conditions, the concentration of the released substance remains higher near the boundary, meaning that the difference in concentration of bioactives between the outside and inside the hydrogels becomes less, resulting in a reduced release rate. External boundary conditions, such as agitation of the release medium, generally enhance the diffusive release of drugs from hydrogel delivery systems by promoting mass transport between the hydrogel and the surrounding medium. Drug release has been reported to vary with agitation speed (e.g., 50, 150, and 250 rpm), with higher speeds accelerating release [128]. Typically, agitation is applied in the range of 50–150 rpm, and a continuous speed of around 100 rpm is recommended, as it approximates the flow conditions encountered in physiological environments such as the bloodstream [30].

Under internal moving boundary conditions, polymeric matrices can experience a swelling-driven phase transition, shifting from a glassy (dry) state to a rubbery (hydrated) state [129,130]. During swelling, a rapidly advancing boundary forms within the shrinking glassy phase, where immobilized microconstituents remain fixed, allowing dissolved bioactive compounds to move through the rubbery phase and be released into the surrounding aqueous medium [131,132]. Therefore, the rate and position of the boundary between the glassy and rubbery regions of the polymer matrix are key determinants of the diffusion kinetics of solutes [81,133].

#### 5.1.2. pH

**Figure 2:**
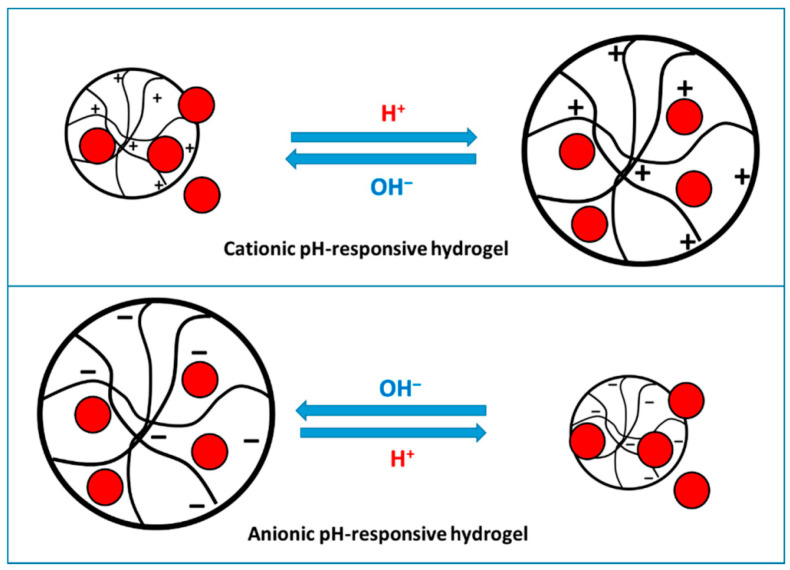
A schematic illustrating the typical behavior of pH-responsive polymeric hydrogels.

The release of bioactive agents is significantly affected by pH through several mechanisms. In particular, pH can modify functional groups, such as carboxyl and amine groups, in polymers like polymethacrylic acid, polyacrylamide, and poly (dimethylaminoethyl methacrylate). Acting as a proton donor or acceptor, pH facilitates the release of bioactive compounds in Figure 2. This process occurs via changes in polymer hydrophobicity, structural deformation, conformational shifts, and polymer dissolution [134].

Cationic hydrogels expand in acidic conditions and contract under basic conditions, releasing their payload, whereas anionic hydrogels behave in the opposite manner, adapted from reference [27].

**Figure 3:**
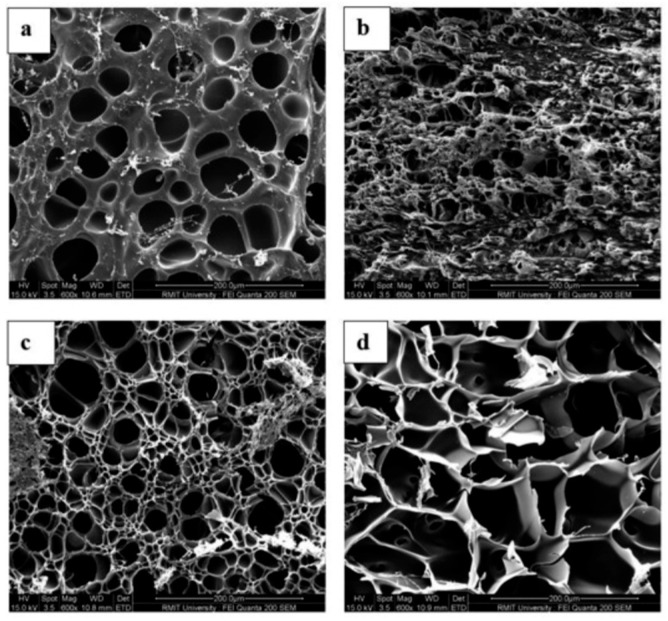
The morphology of BSA networks crosslinked with 0.5 g/100 g genipin was visualized by SEM at pH (**a**) 2.0, (**b**) 4.8, (**c**) 7.0, and (**d**) 11, using 200× magnification at 24 °C, adapted from reference [136].

Changing the pH from 5.5 to 8.5 led to an increase in the cumulative release of naringenin from carboxymethyl cellulose/2-hydroxyethyl acrylate, rising from 42% to 73% [135]. he release of β-carotene from genipin-crosslinked K-carrageenan/carboxymethyl cellulose beads was approximately 2.5 times lower in acidic buffer (pH 1.2) compared to near-neutral conditions (pH 7.4) [136]. At a pH 7.4, the beads exhibited swelling as a result of the electrostatic repulsion among the ionized carboxyl groups of carboxymethyl cellulose, which engage in hydrogen bonding under acidic conditions. Conversely, a different trend was noted regarding the release of lysozyme from gelatin/genipin films [137]. Lysozyme was released rapidly under acidic conditions (pH 3.8) compared to neutral pH (7.0). This burst release from the composite system was linked to film degradation, triggered by acid hydrolysis of gelatin networks and the breakdown of genipin–gelatin crosslinks at low pH. Similarly, lowering the pH from 7.4 to 3.0 increased the release of curcumin from 1,3-bis(N,N-dimethyl-N-cetylammonium)-2-propyl acrylate dibromide, which was attributed to reduced electrostatic interactions due to protonation of functional groups [138], whereas curcumin release from alginate hydrogel nanoemulsions was significantly higher under alkaline conditions (pH 9.0), due to accelerated degradation of sodium alginate gel beads [139]. The structure of genipin-crosslinked BSA networks was examined using scanning electron microscopy (SEM), as illustrated in Figure 3. The swelling at alkaline pH levels resulted in changes to the structural characteristics of the BSA matrix. A qualitative correlation was observed between the progressively more open structures of the protein matrix, as indicated by SEM, and the decline in mechanical properties noted through rheological measurements [140].

This pH sensitivity was utilized in entrapping and releasing of probiotics from ethylenediaminetetraacetic-calcium-alginate (EDTA-Ca-Alg) system. In an acidic environment, the hydrogel maintained a compact structure with minimal pore formation, ensuring the protection of probiotics, *L. rhamnosus* ATCC 53103. Conversely, in a neutral intestinal environment, the hydrogel structure progressively disintegrated due to the release of Ca^2+^ from the hydrogel, which led to the release of cells [109]. Similarly, isoquercitin (IQ) loaded okra polysaccharides/gelatin complex coacervates (OGIQ) demonstrated pH responsiveness and targeted delivery to the intestine. The release rate of IQ from OGIQ was measured at 89.4% in intestinal fluid, while it remained below 2% during acidic and simulated gastric digestion [102].

Indeed, among the various chemical factors, the influence of pH stands out as a critical determinant in the release of bioactive substances [141]. It acts as an independent regulator of release behavior by altering the ionization state of functional groups, polymer conformation, and network integrity. For instance, in gelatin-based hydrogels, low pH conditions can trigger burst release due to acid-induced hydrolysis of the gelatin. Conversely, in BSA based hydrogels, alkaline conditions lead to swelling and structural degradation, which progressively opens the network and facilitates diffusion. These observations collectively demonstrate that pH-driven structural transitions directly govern the release mechanism. Additionally, pH acts synergistically with other factors, including ionic strength. The electrostatic interactions between carrier and entrapped bioactive compound, which ultimately govern its release, are significantly influenced by pH levels.

#### 5.1.3. Ionic Profile

The ionic properties of the release medium can facilitate the diffusion of bioactive compounds by promoting the loosening of crosslinking points within the biopolymer network or by accelerating matrix erosion [142,143,144]. Ions in the release medium can significantly affect the release rate of bioactive compounds. In cases where bioactive molecules interact with polymers through coordinative bonds, the presence of ions or competing ligands may alter these interactions. To evaluate the bioaccessibility and release of vitamin E encapsulated in oil-in-water emulsions, a simulated small intestinal fluid was employed. The results showed that the addition of calcium ions enhanced the release rate of vitamin E in this system [145]. The ionic strength influences the expansion of polymers containing ionizable groups by modifying their solubility and swelling characteristics. In contrast, nonionizable polymers remain unaffected by changes in ionic strength. When the ionic strength exceeds a certain level, complexes can dissociate as a result of reduced electrostatic interactions, a process referred to as the electrostatic screening effect. Additionally, the swelling behavior of hydrogels made from sodium carboxymethyl cellulose is influenced by the ionic strength of the surrounding medium [146].

In vitro drug release from biopolymeric matrices is influenced not only by the pH and ionic strength of the dissolution medium but also by the presence of electrolytes [147]. The effect of the dissolution medium on the in vitro release of drugs from ionically gelled pectinate beads was investigated. It was observed that rutin was released more rapidly from calcium pectinate beads when phosphate buffers were used, likely due to the formation of CaHPO_4_ precipitate, which can create a “pumping” effect on calcium ions, potentially disrupting the gel network and enhancing rutin release. In the case of zinc pectinate beads, two distinct precipitates may form depending on the electrolyte composition of the medium. The formation of Zn_3_(PO_4_)_2_, which can coat the beads, may slow rutin release in Sorensen’s buffer, whereas the generation of ZnHPO_4_ could promote zinc ion release and thereby increase rutin release in a citrate-phosphate buffer [148]. Another study was conducted to evaluate the effect of the conditions of release medium changing bilayer numbers of the matrix, temperature, pH, and ionic strength. It was observed that bioactive release (tannic acid) from antioxidative cellulose nanofiber films (polyethylene glycol/tannic acid films) was found to increase with a higher number of bilayers, as well as with elevated pH and temperature; however, it decreased with an increase in ionic strength [122].

#### 5.1.4. Nature of Solvent

The capacity of the solvent to dissolve the bioactive compound is essential for its liberation from the hydrogel. It is important that the polarity of the solvent and bioactive compound is compatible. Polar bioactives tend to dissolve better in polar solvents (like water), while non-polar bioactives prefer non-polar solvents. The solvent’s interaction with the hydrogel polymer matrix is also important. The release kinetics of a bioactive compound are influenced by the ability of the solvent to penetrate the hydrogel matrix and by the partitioning behavior of the bioactive between the solvent and the hydrogel [149]. Several studies have shown that the polarity of the surrounding medium can affect the release of bioactive compounds from different carrier systems. For example, the release behavior of gallic acid encapsulated in poly(lactic acid) nanofibers was assessed in aqueous ethanol solutions at concentrations of 10% and 95% [150]. Due to the hydrophilic nature of gallic acid, its highest release rates were observed in both pure aqueous and 10% ethanol solutions. Similarly, the release behavior of oregano essential oil and green tea extract encapsulated in ethylene-PVA films was studied in 3% acetic acid, 10% ethanol, and 50% ethanol solutions. The films demonstrated the greatest antioxidant release in the 50% ethanol medium [151]. The release behavior of polyphenols from starch–chitosan films is strongly influenced by both the pH and polarity of the solvent [151], such that fewer polyphenols are released under acidic conditions, and the release occurs at a slower rate compared to both hydrophilic and lipophilic environments.

A high diffusion rate of bioactive compounds in low-polarity media has also been reported for carvacrol released from poly(ε-caprolactone) [152], and catechin (tea polyphenol) from chitosan-PVA film [153]. In contrast, The rate of release of rosemary polyphenols into ethanol, which serves as a fatty food simulant, was observed to be slower in comparison to the release into an aqueous medium [154]. Comparable results were reported in a study investigating the release of *Mentha longifolia* L. essential oil from Balangu seed gum–polyvinyl alcohol (PVA) nanocapsules in different media, including distilled water, 10% ethanol, 50% ethanol, and 3% acetic acid. The essential oil exhibited the highest release in distilled water, followed sequentially by 10% ethanol, 50% ethanol, and 3% acetic acid. The study also highlighted the considerable solubility and swelling ability of Balangu seed gum and PVA in water, whereas both components were poorly soluble in ethanol and acetic acid, with the gum being only slightly soluble in acetic acid [155]. An opposite trend was observed in another investigation, where the release kinetics of polyphenols from thyme extract (TE) incorporated in chitosan (CH) films were assessed using three different solvents: acetic acid solution, distilled water, and ethanol. The highest quantity of polyphenols was observed in the acetic acid solution, attributed to the enhanced solubility of chitosan. Consequently, the polarity of the solvents significantly influenced the release of polyphenols from the films [116].

#### 5.1.5. Temperature

Temperature serves as a crucial physical environmental factor. It is widely recognized that variations in temperature significantly influence molecular motion. The diffusion process accelerates with an increase in temperature, as structural relaxation generates voids within the matrix, facilitating the swift movement of penetrants through these channels [156]. Initial research on breakfast cereals has shown a significant correlation between water absorption and temperature increase, consistent with the principles outlined in the Arrhenius law [157,158]. The glass transition temperature serves as a useful indicator for observing diffusion processes. As temperature rises and approaches the glass transition zone, there is an increase in the available voids, which facilitates the movement of diffusing component [156,159]. Rising temperatures supply thermal energy that enables the overcoming of attractive forces between polymer chains and bioactive molecules, thereby improving transport phenomena. In an investigation, novel stimuli-responsive hybrid hydrogels were developed using succinylated cellulose nanocrystals (Su-CNC). The innovation involved incorporating Su-CNC with varying degrees of substitution (DS) into the hydrogel matrix, enabling it to exhibit pH and thermal responsiveness through a free radical polymerization reaction with poly(N-isopropylacrylamide) (PNIPAm). It demonstrated a significant response to temperature variations, as its swelling behaviour and hydrophilicity decreased at temperatures of 35 °C and above. This change resulted in increased hydrophobicity and subsequent hydrogel contraction [123]. To observe the effect of temperature on release profile, an investigation was conducted on an Amazonian fruit named camu-camu. The fruit extract, including both pulp and peel, was microencapsulated via spray drying using maltodextrin, inulin, and oligofructose as carriers. The resulting microcapsules were evaluated for their physicochemical and thermal properties, as well as their controlled release behavior at different temperatures (25 °C and 35 °C). Increasing the temperature from 25 °C to 35 °C led to an enhanced release of bioactive compounds in all formulations [121]. Thermo-responsive hydrogels alter their hydrophilic–hydrophobic balance at the lower critical solution temperature (LCST); for example, PNIPAm has an LCST of ~32 °C. Bioactive release increases below the LCST due to swelling and thermal activation, but decreases above it as the hydrogel’s hydrophilic character diminishes with the loss of intermolecular hydrogen bonds.

### 5.2. Hydrogel Properties

In a delivery system, natural bioactives are entrapped within different biopolymers, which significantly influence the release rate of these entrapped compounds according to requirement.

#### 5.2.1. Polymer Types

Various biopolymers, such as carbohydrates, proteins, and their blends, can be employed to develop delivery systems for different bioactive compounds. The structural characteristics of biomaterials, built by molecular organization and concentration, play a vital role in the diffusion of bioactives from either individual or composite formulations. Material composites are typically favoured for their ability to provide the necessary techno-functionality that supports bio-functionality through the regulation of drug release. The combination of natural polymers leads to the formation of binary systems characterized by either a phase-separated or an associated network structure [160]. Polymers of lower molecular weight enhance the available free volume within the matrix, reduce the activation energy required for conformational changes between states, and promote faster diffusion kinetics. In contrast, higher molecular weight polymers form entanglements that resemble a cage-like structure, thereby limiting the mobility of diffusing substances [161,162]. In vitro studies showed that β-carotene encapsulated in zein protein was rapidly released in the gastric environment, due to the positive charge of zein and protonation of β-carotene, which weakened their interaction.

In contrast, the presence of pepsin leads to the degradation of zein, weakening its structure and facilitating the release of β-carotene in the gastric environment. On the other hand, encapsulation of β-carotene using whey protein isolates and gum acacia produced a more sustained release during the gastric phase, due to the protective effect of these wall materials [163,164,165]. Curcumin encapsulated in Lactoferrin/pectin polyelectrolyte complex nanoparticles (PEC NPs) showed rapid release under acidic conditions. This phenomenon can be attributed to the electrostatic interactions among the carrier components, which lead to a disruption in the structure of the PEC NPs, resulting in a swift release. Conversely, curcumin encapsulated with chitosan and gum Arabic demonstrated a slower release in the gastric environment. This behavior suggests that the polymer matrix has a more stable structure and greater swelling capacity, attributed to the amino groups present in chitosan [166,167].

#### 5.2.2. Polymer Crosslinking

The strategy of employing proteins in carrier-mediated transport presents promising opportunities for advancements in biology and chemistry, particularly in the realm of drug delivery. However, the unavoidable infiltration of water molecules results in network degradation and an unintended rapid release of the drug, which should be prevented, as it diminishes the treatment’s effectiveness [168,169]. Crosslinking is considered a valuable strategy for designing delivery systems that can achieve tailored release kinetics for specific therapeutic needs [170]. Cross-linking is a polymer chemistry technique that improves the stability of polymer chains by creating a network structure through multidimensional connections. A cross-link is a bond that links one polymer chain to another, which may be either covalent or ionic. This process converts a liquid polymer into a gel or solid state by restricting chain mobility. Moreover, cross-linking leads to an increase in the overall molecular weight of the polymer [171]. Crosslinking agents are generally classified into two categories: chemical and physical crosslinkers.

##### Chemical Crosslinking

A summary of chemical crosslinkers used in polymeric matrices has been listed in Table 3. Crosslinking agents that are suitable for oral delivery include genipin, transglutaminase, anhydrous tripolyphosphate, glutaraldehyde, and citric acid. These agents have been used to alter the structural characteristics of delivery systems by either forming interactions with oppositely charged polymer chains or through enzyme-mediated crosslinking mechanisms [29]. However, the production of hydrogels for biomaterial applications necessitates meticulous planning and the appropriate choice of the crosslinking method. This process significantly influences the chemical and mechanical characteristics of the hydrogel, which in turn impacts the cellular responses elicited by the hydrogel. The selection process must consider factors such as solubility, reaction conditions (including pH and temperature), potential side reactions, and the overall reactivity. Depending on the intended use, it is essential to evaluate the crosslinking rate, density, hydrogel stability, and the biocompatibility of both the crosslinker and the crosslinked biopolymers [172]. Chemical crosslinkers that exhibit cytotoxicity, such as glutaraldehyde [173], can raise cytocompatibility concerns, whereas genipin exhibits much lower toxicity [174]. A low level of crosslinking may lead to a hydrogel exhibiting poor mechanical strength and stability characteristics, while an excessive degree of crosslinking can cause reduced porosity, adversely affecting cell viability and biodegradation [172].

Genipin is a non-toxic crosslinking agent increasingly used in functional foods and pharmaceutical formulations. In the presence of oxygen, it reacts with lysine residues from neighboring protein chains, forming stable crosslinked structures [230]. In contrast to traditional chemical cross-linkers such as formaldehyde and glutaraldehyde, genipin offers several safety benefits, including anti-inflammatory, anticancer, and antibacterial properties. Consequently, genipin has been the subject of extensive research over recent decades, establishing itself as a safe and efficient chemical cross-linker across a wide range of delivery systems [29]. Genipin has been demonstrated to effectively limit the burst release of entrapped agent while prolonging the release duration [207]. It enhanced the stability of hydrogels under acidic conditions by reducing the availability of free amino groups in the polymer chain necessary for ionization, which led to a decreased swelling ratio and extent of release [193,196,202], especially a lower release rate at higher pH (pH 8), and a higher release rate in lower pH (pH 1.5) [209]. Moreover, genipin enhanced resistance to proteolytic degradation, thereby decreasing curcumin release in the gastric environment [203]. Compared with glutaraldehyde-crosslinked gels, genipin-crosslinked chitosan hydrogels exhibited lower swelling and more controlled release due to their greater rigidity and stability [205]. Furthermore, genipin was found to be effective in the entrapment and sustained release of vitamin B6 [80,81,140], n-3 fatty acids [210], lidocaine [192], and vitamins C and E [82,199], among others.

The enzymatic approach to crosslinking offers advantages including efficient catalysis, gentle reaction conditions, and non-toxicity. Hydrogels prepared enzymatically are highly biocompatible and degradable, positioning them as promising materials for use in food processing, tissue engineering, sustained-release formulations, bioplastics, and biosensing devices [231,232]. Derived from *Streptoverticillium mobaraense*, microbial transglutaminase (mTGase) is a transferase that promotes inter- and intramolecular crosslinking through amide bond formation between the carboxyamide moieties of acyl donors and lysine amino groups of acyl acceptors [233,234]. Microbial transglutaminase was employed to crosslink bitter apricot kernel protein, enabling a controlled release of riboflavin. The results indicated that the enzyme successfully generated crosslinks that increased the gel’s hardness, elasticity, and cohesiveness, leading to a more sustained release profile. This formulation was identified as a promising carrier for protecting sensitive bioactive molecules, with potential utility in both food and pharmaceutical applications [176]. In other studies, transglutaminase crosslinking was found to be a promising approach for biopolymeric gels, offering improved stability, regulated release, and an enhanced system for delivering bioactive compounds [175,177,179,182]. However, it is essential to screen and assess cross-linking enzymes capable of crosslinking various substrates, given the extensive range and diversity of these substrates. Comprehensive research into the molecular level crosslinking mechanisms of enzymes and their substrates is also essential to establish a theoretical foundation for enhancing the properties of hydrogels.

Glutaraldehyde is widely used as a crosslinking agent in the preparation of polymer-based particles, films, and fibers designed for controlled delivery applications. Numerous investigations have shown that glutaraldehyde-crosslinked biopolymer matrices can modulate the release of therapeutic and bioactive compounds. In one study, post-treatment with glutaraldehyde (GTA) vapors was applied to electrospun zein nanofibers loaded with eugenol to improve their physicochemical properties and release behavior. Relative to the uncrosslinked fibers, the crosslinked systems preserved their fibrous morphology but exhibited increased fiber diameter and a denser structure. Achieving an appropriate crosslinking level was essential for enhancing the stability and mechanical integrity of the zein network. The GTA vapor-treated nanofibers provided a controlled release of eugenol in phosphate-buffered saline (PBS), demonstrating strong potential for enriched food products as well as smart or active food packaging applications [183].

In a similar vein, other researchers have documented comparable findings regarding the development of chitosan microspheres [184,185,187,190], gelatin nanoparticles [186,188], and silk fibroin film [189] intended for the delivery and controlled release of functional agents, utilizing glutaraldehyde as a crosslinking agent. Whey protein-based microcapsules were synthesized utilizing glutaraldehyde. Casein microspheres crosslinked with glutaraldehyde were shown to function effectively as carriers capable of regulating the diffusion of bioactive compounds. At pH 7.4, the microspheres exhibited enhanced pore development, resulting in greater swelling and consequently facilitating drug diffusion [235]. Similarly, the release observed under simulated intestinal conditions exceeded that occurred under simulated gastric conditions [236]. Although numerous studies have examined in vitro release profiles, far fewer have evaluated in vitro functional activities—such as antioxidant and antimicrobial effects, microbial penetration, and cell cytotoxicity—or conducted in vivo assessments of crosslinked delivery systems.

##### Dual Crosslinking

In certain instances, the use of dual crosslinkers improves the effectiveness of the crosslinking process, thereby enhancing the overall release profile of bioactive compounds from the matrices. A research investigation focused on the development of gelatin-chitosan microcapsules utilizing a dual cross-linking method involving transglutaminase (TGase) and tannic acid (TA). The application of dual cross-linking resulted in improved structural integrity of the microcapsules. The presence of TA allowed the dual cross-linked microcapsules to demonstrate superior controlled-release characteristics compared to those that were single cross-linked, as evidenced by a reduced cumulative release rate [227]. Likewise, studies on bovine serum albumin gels treated with both microbial transglutaminase and ribose to modulate caffeine release showed that the dual-crosslinking approach was more effective in simulated saliva and gastric fluids [237]. The comparable results were observed in other research studies [198,215,225,226].

Hydrogel crosslinking can occur via covalent or non-covalent interactions between polymer chains. Covalent bonds provide energetic stability, producing mechanically robust networks, while non-covalent interactions, such as hydrogen bonding, are sensitive to environmental factors like pH and temperature, influencing hydrogel formation, stability, and functional properties. By combining covalent and non-covalent mechanisms—i.e., using dual crosslinkers—it is possible to fine-tune network structure, mechanical strength, and release behavior, offering a versatile design strategy to optimize hydrogel performance. Overall, reported successful dual-crosslinking systems include transglutaminase–tannic acid, transglutaminase–ribose, genipin–disulfide, citric acid–calcium chloride, tripolyphosphate–dextran sulfate, and epichlorohydrin–calcium chloride–tannic acid.

##### Physical Crosslinking

A significant transformation has taken place in the food processing industry with the introduction of advanced non-thermal technologies, which have enhanced the quality, safety, and functionality of processed foods. Non-thermal methods, such as gamma radiation, ultrasound, and near-infrared techniques, utilize mechanical waves and the phenomenon of acoustic cavitation to rapidly alter physical structures, thereby promoting chemical reactions in proteins that affect their physicochemical and functional characteristics [238]. Physical crosslinking approaches help control the release of bioactive compounds by generating additional crosslinks—formed through interactions between atoms—and also improve the technological and functional properties of biopolymers by reinforcing their physical and mechanical structure. The research conducted in this area is compiled in Table 4.

Protein matrices can be formed through physical crosslinking methods, such as gamma irradiation and dehydrothermal treatment, which rely on various non-covalent interactions including ionic bonds, hydrogen bonding, van der Waals forces, and hydrophobic interactions. The effect of electron beam irradiation on fish gelatin films containing bamboo leaf antioxidants was investigated, showing that irradiation enhanced tensile strength, opacity, denaturation temperature, and microstructural properties. Infrared spectroscopy and microstructural analyses suggested that appropriate irradiation doses promoted the formation of crosslinking interactions between gelatin and the antioxidants, resulting in compact structures that slowed antioxidant release [243]. In a fibrous chitosan–curcumin polymer, exposure to 1 Gy of gamma irradiation resulted in the release of 5 ± 1% of the conjugated curcumin, whereas exposure to 6 Gy led to the release of 98 ± 1% of the conjugated curcumin [108,126,153,241]. Similarly, various physical crosslinking treatments, such as ultrasound [228], near-infrared [127], ultraviolet radiation [111], and microwave [248] demonstrated improved mechanical and release properties of crosslinked polymeric structures.

A combination of gelatin and phenylazide-conjugated poly(acrylic acid) was subjected to electrospinning under ultraviolet light. In contrast to the conventional crosslinking method utilizing glutaraldehyde vapor, the gelatin electrospun fibres (GESFs) that underwent UV crosslinking exhibited enhanced characteristics, including improved preservation of GESF morphology, consistent crosslinking throughout the fibres, reduced cytotoxicity, and maintained bio-functionality. L929 cells demonstrated superior growth on the UV-crosslinked GESF scaffolds in comparison to those crosslinked with glutaraldehyde [246]. In contrast, The films made from chemically (glutaraldehyde) crosslinked methylcellulose exhibited a homogeneous gel structure, while those produced from radiation crosslinked methylcellulose displayed a less uniform crosslinked composition [252].

Physical crosslinking treatments can accelerate the chemical crosslinking process. Vanillin cross-linked chitosan was synthesized using both the refluxing and microwave irradiation methods, which were subsequently compared. The refluxing method necessitated a reaction time of 6 h, while the microwave irradiation method achieved completion in just 1 to 5 min [224]. The sustained release of drug from a microwave-assisted pH-responsive hybrid polymer network, which consists of chitosan and gelatin cross-linked with glutaraldehyde, was documented by [103]. However, without microwave assistance, the same behaviour was not observed. That is why, in depth comparative analysis is needed to fill up the gaps.

In summary, chemical crosslinkers offer the advantage of easily designing and precisely controlling hydrogel release profiles over time. Naturally derived crosslinkers, such as microbial transglutaminase and genipin, serve as safer alternatives to conventional chemical crosslinkers. Moreover, the use of dual crosslinkers, combining complementary mechanisms, can further enhance network stability, mechanical strength, and tunable release behavior.

#### 5.2.3. Effect of Plasticisers

Plasticizers are frequently incorporated into biopolymeric films to overcome certain limitations. These substances (outlined in Table 5) enhance macromolecules’ mobility by decreasing the interconnections between polymer chains.

These additives increase the intermolecular spacing, or free volume, similar to the effect of temperature, and act as “mobility enhancers” that ultimately lower Tg [265,266,267]. Plasticisers also reduce the melting temperatures and crystallization [267]. The distinct characteristics of plasticisers contribute to an increased presence of the amorphous phase in the intermolecular structure of polymers. The integration of plasticisers with polymers enhances the ionic conductivity of polymeric films, all the while preserving their flexibility and thermal stability [268]. Findings from reference [269] indicate that an increase in plasticiser concentration resulted in a reduction in melting temperature and an enhancement of crystallinity in poly(3-hydroxybutyrate-co-3-hydroxyvalerate) (PHBV) when various types and concentrations of plasticisers were employed to enhance flexibility and elongation. The plasticizers evaluated in the study included propylene glycol, glycerol, triethyl citrate, castor oil, epoxidized soybean oil, and polyethylene glycol. It was found that medium molecular weight compounds possessing ether or ketone functionalities, which can interact effectively with PHBV, acted as the most efficient plasticizers. In contrast, long side chains appeared to hinder the interaction of ketone or hydroxyl groups with the PHBV matrix, while low molecular weight compounds containing hydroxyl groups showed negligible plasticizing effects. Among the tested agents, triethyl citrate and polyethylene glycol produced the most favorable thermal, mechanical, and barrier properties. These findings suggest that further research focusing on plasticizer selection based on their physicochemical characteristics may be beneficial.

In a separate investigation, it was observed that glycerol and sorbitol diminished the intermolecular forces between neighbouring chain segments, thereby increasing the spacing between them. This modification allowed water molecules to penetrate the more hydrated, solid-like matrices and supported the diffusion of small bioactive compounds into the surrounding medium [270]. Thus, the transport of potassium sorbate from chitosan films increased four times when palmitic acid was present. The improved diffusion was ascribed to the formation of voids within the films, arising from the multilayered architecture of the lipid–chitosan emulsion. Substantial swelling of the matrix, reaching roughly 40% of a single chitosan film’s thickness, allowed potassium sorbate to migrate freely through the chitosan network [271]. Another study explored the influence of plasticizers, specifically sorbitol and Tween 80 (T80), on the drug release behavior of rice starch wafers. Sorbitol produced a more compact structure, exhibiting higher puncture strength (PS) but lower water absorption capacity (WAC), whereas T80 led to a looser matrix, decreasing PS and increasing WAC. The smaller molecular size of sorbitol likely enabled stronger interactions with starch molecules, resulting in a denser wafer structure. This rigid network also contributed to reduced WAC and a slower, more sustained drug release [272].

#### 5.2.4. Swelling and Microstructural Properties

Understanding the swelling behavior in food-based systems allows precise control over mechanical properties and diffusion rates, facilitating targeted delivery for a variety of biomedical and therapeutic applications [82]. he movement of entrapped molecules is subsequently affected by both the volume and rate of water penetrating the matrix. The swelling behavior of hydrogels is determined by factors such as crosslinking density, network mesh size, wall thickness, and the presence of hydrophilic functional groups within the gel [273]. Hydrogels with lower chemical crosslinking exhibited greater volumetric changes, which took place over shorter time intervals [84]. These distinct swelling properties likely result from the strong chemical crosslinks formed by genipin between gelatin chains, unlike the temporary physical interactions present in other matrices [171]. Interestingly, the results also indicated that incorporating bioactive compounds had little effect on the swelling behavior of the matrices. Instead, the extent of swelling was primarily governed by the concentration of genipin [92,274].

Although scanning electron microscopy imaging offers a topographical perspective of the superficial pores of the gel (Figure 3), the diameter of the mesh size can be estimated by applying the Flory-Rehner theory in conjunction with swelling measurements. Key parameters obtained from the swelling analysis include the polymer volume fraction, the molecular weight between crosslinks, the crosslink density, and the network mesh size of the polymer [81]. With increasing swelling, conventional triple-helix structures form through hydrogen bonding with water molecules, accompanied by the creation of chemical crosslinks. This leads to an apparent increase in the molecular weight between crosslinks. This phenomenon persists until the state of equilibrium swelling is achieved. The size of the mesh also increases in a comparable way, as the infusion of water molecules causes the voids between neighbouring chain segments to expand [82]. Because Flory–Rehner parameters directly determine network porosity, they also govern drug release kinetics: larger mesh sizes allow faster diffusion-driven release, whereas smaller meshes restrict molecular mobility and shift release toward relaxation- or erosion-controlled mechanisms. Thus, the structural parameters predicted by Flory–Rehner theory provide a quantitative basis for interpreting and predicting hydrogel release behavior. In a system crosslinked with 0.1% genipin, the initial measurements for polymer volume fraction, molecular weight between crosslinks, crosslink density, and mesh size were 0.901, 8.6 g/mol, 8985, and 3.22 nm, respectively, which changed to 0.756, 25.9 g/mol, 2904, and 5.97 nm after 24 h of immersion [84]. Swelling also decreases the total solids content of the system as water molecules are absorbed from the initial dry state of 93% (*w*/*w*) total solids to 42% (*w*/*w*), reaching equilibrium swelling after approximately 48 h (2900 min) [82]. It is noteworthy that the absorption of water during the swelling process does not interfere with the covalent bonds between genipin and gelatin molecules. This swelling causes the overall volume of the sample to expand, thereby decreasing the crosslink density, which represents the number of crosslinks per unit volume.

Within the gel system, the polymer volume fraction influences properties such as mechanical stability, opacity, and permeability. Increasing the amount of genipin crosslinker raises the polymer volume fraction by creating a more compact structure that restricts water uptake [275]. With higher polymer concentrations in the hydrogel, the release of bioactive compounds slows down, as their diffusion paths become more convoluted, requiring adjustments to the diffusion coefficients to account for increased tortuosity [276]. The swollen polymer volume fraction is determined by factors such as initial polymer content, molecular weight, branching, charge density, flexibility, crosslink type and density, configuration, and solvent–polymer interactions [277]. Raising the genipin concentration in BSA networks from 0.5% to 4% (*w*/*w*) resulted in a marked decrease in the swelling ratio, from 14.7 to 0.2, while the polymer volume fraction increased from 0.1 to 0.85. This denser network structure consequently slowed the release of the encapsulated vitamin B6 [80]. For the hydrogel crosslinked with 0.1% genipin, the polymer volume fraction declined from 0.901 to 0.756 over 1440 min, whereas a higher genipin concentration of 2% resulted in a decrease from 0.971 to 0.803 [84].

The mesh size, or correlation length (ξ), represents the linear distance between neighboring crosslinks and has been identified as a key structural factor influencing the controlled release of bioactive compounds from swellable carriers [278]. Most frequently, hydrogel mesh size is determined utilizing equilibrium swelling theory (i.e., Flory–Rehner) [279] or rubber elasticity theory [280], although the Mackintosh theory [281], the blob model [282], NMR [283], small-angle X-ray scattering [284], small angle neutron scattering [285], and correlations based on dextran diffusion [286] have also been used with success. The mesh size can be adjusted to regulate the controlled release of bioactive compounds, depending on factors such as the polymer type and concentration, the nature or extent of crosslinking, and external stimuli including pH, ionic strength, and temperature [277,287]. Increasing the genipin crosslinking concentration resulted in smaller mesh sizes, with values decreasing from 5.97 nm at 0.1% genipin to 3.97 nm at 2%, as stronger crosslinking drew gelatin polymer chains closer together [84]. Gelatin networks crosslinked at 25 °C exhibited pore sizes below 50 μm, while crosslinking at lower temperatures produced less compact structures with larger pores [288]. At neutral pH, the release of vitamin B6 was markedly slowed due to a reduction in mesh size from 59.1 to 1.1 nm, whereas at alkaline pH (11.0), the protein network’s molecular pore size expanded, facilitating the release of vitamin B6 into the surrounding medium [80].

### 5.3. Characteristics of Bioactive Compounds

In addition to the matrix density, crosslinking, and molecular interactions between the polymer network and the bioactive agent discussed earlier, the morphological characteristics of penetrant molecules—such as size, shape, conformational chemistry, and ionic nature—also significantly influence the behavior of delivery vehicles. The ability of a penetrant to diffuse into the polymeric matrix depends on both these interactions and the available free space, and this step often becomes the rate-limiting factor in the release of the bioactive compound [289]. The outcome can be either beneficial or detrimental, depending on the properties of the active compound and the specific biopolymers used as the matrix [35].

Penetrant compounds can enhance the physical and functional characteristics of edible films by facilitating the formation of cross-links among proteins and/or polysaccharides [290,291]. Furthermore, modifying the film structure via these cross-linking reactions can affect the retention of active compounds within the polymer network, thereby enabling controlled adjustments in their release [241,292]. Ascorbic acid was identified as a plasticizer for films made from potato starch and polyvinyl alcohol (PVA). The addition of ascorbic acid at a concentration of 20% resulted in a 35% reduction in the tensile strength (TS) of the PVA-based films, while simultaneously increasing the elongation at break by a factor of five [293]. The presence of functional groups in ascorbic acid facilitates a more effective interaction between starch and PVA molecules, potentially improving their mechanical properties. In contrast, the incorporation of tartaric acid at 1% *w*/*w* of dry matter into carboxymethylcellulose films did not exhibit any anti-plasticizing or crosslinking effects, nor did it enhance the mechanical properties of the films. This lack of effect may be attributed to the insufficient formation or absence of chemical bonds at this concentration of organic acid. In a separate study, the addition of lysozyme was frequently found to adversely affect the mechanical properties of edible films and coatings, primarily due to its degradative impact on polymer chains, particularly in protein-based films. Specifically, the tensile strength (TS) and elongation at break (EAB) of chitosan films decreased with increasing lysozyme concentration [294].

When the particle size of the solute exceeds the mesh size of the polymer network, the solute cannot penetrate the matrix; this phenomenon is commonly referred to as the “screening effect” of the polymer [161]. Evidence of this phenomenon was observed in genipin-crosslinked casein systems during the preparation of “drug” formulations, where the infusion of a BSA solution into the casein xerogel was hindered due to the polymer network’s screening effect [295]. The movement of large penetrant molecules can be regulated through chain disentanglement, structural relaxation, or swelling of the matrix, which effectively increases the network’s “apparent pore size” and enhances its utility [296]. Recently, a theoretical approach has been presented that predicts the diffusion coefficients of solutes diffusing from genipin crosslinked gelatin matrices [297]. This model showed a linear correlation between diffusion coefficient and molecular size of hydrophilic diffusants. The diffusants used were small molecules, with molecular weights ranging from 58.44 to 342 g/mol. Therefore, investigating diffusants with higher molecular weights could offer valuable insights into the influence of solute size on diffusion and holds strong potential for further exploration.

The shape of the penetrant molecule also exerts a significant influence on transport phenomena [298]. The atomic arrangement of molecules, which determines their well-defined morphology, influences their binding capacity to neighboring penetrant molecules and/or polymeric network chains [299].

## 6. Conclusions and Prospects

For the sustained release and targeted delivery of bioactive compounds, it is essential to comprehend the mechanisms of digestion and absorption of these compounds before creating suitable carriers for their transport. It is crucial for carriers to uphold stability in the face of adverse environmental conditions and to prevent the premature release of entrapped compounds. Additionally, solute must possess the capability to diffuse effectively into the targeted area and release the bioactive compounds as required. Various strategies were proposed to create resilient structures that can release bioactives in a controlled and preferred manner. To effectively manage the release of bioactive compounds from various structures, it is essential to consider several critical factors, including the properties of the release medium, the characteristics of the hydrogel, and the inherent properties of the bioactive compounds themselves. The release conditions of media, particularly pH, ionic properties, temperature, and solvent characteristics, play a vital role in the liberation of bioactive substances. These factors influence the functional groups, the electrostatic interactions between the carrier and the encapsulated bioactive compound, as well as the dissociation and conformational alterations of polymers.

The properties of delivery systems can be modulated through the selection of polymers, cross-linkers, plasticizers, and specific environmental conditions. Despite the considerable volume of research on release profiles, there remains a significant shortage of studies examining the functional activities of delivery systems crosslinked by chemical crosslinkers, including antioxidant and antimicrobial properties, microbial penetration assays, and cell cytotoxicity evaluations. Also, in-depth investigation into the crosslinking mechanism in molecular level between enzymic crosslinkers and their substrates is crucial for developing a theoretical framework aimed at improving the characteristics of hydrogels. Comparative investigations of crosslinkers, both single and multiple, remain inadequately documented. Therefore, a thorough comparative analysis would be beneficial in addressing these gaps. Furthermore, a critical gap exists in understanding the in vivo degradation rate of crosslinked hydrogels and its direct impact on the release mechanism—diffusion-controlled versus erosion-controlled. Future research could explore the development of smart, stimuli-responsive hydrogels to achieve more precise and controlled delivery of bioactive compounds.

## Figures and Tables

**Table 1:** A summary of the mechanisms of absorption and the environmental factors that affect bioactive compounds.

Bioactive Compounds	Environmental Influence Factors	Absorption Mechanisms in Small Intestine	References
vitamin A	Light, Acid, Heat, Humidity	Retinol absorption may occur through active or passive mechanisms, and fatty meals can increase its uptake.	[36,37]
vitamin B_12_	Light, Heat, pH	Involve three transport protein: intrinsic factor (IF), haptocorrin (HC) and transcobalamin (TC) and their respective receptors.	[38,39]
vitamin C	Heat, Light, Humidity	Na^+^-dependent transporter transportation	[40,41]
vitamin D	Light, pH, hydrophobicity	Passive diffusion and/or receptor-mediated transport	[42,43]
vitamin E	Heat, Light, Humidity	Passive diffusion, Receptor-mediated transport	[44]
β-Carotene	Light, Heat	By passive diffusion and/or transporter-mediated processes	[36,37]
w-3 fatty acid	Heat, Light	Carrier-mediated transport	[45]
Quercetin	Heat, Alkali	Passive diffusion	[46,47]
Caffeine	Temperature	80% of caffeine is absorbed through the gastrointestinal trac, the remaining 20% is absorbed by the stomach.	[48,49]
Astragals polysaccharides	Stickiness, Low solubility	Carrier-mediated transport, Passive diffusion	[50]
Fungal polysaccharides	Heat, Light, Humidity	Clathrin-mediated endocytosis	[51]
Resveratrol	Heat, Light	Passive diffusion and/or transporter-mediated processes	[52]
Ferulic acid	Low solubility, pH, pKa,	Passive transcellular diffusion, monocarboxylic acid transporter (MCT) and an H^+^-dependent carrier	[53,54]

**Table 2:** Effect of release media conditions on the release rate of bioactive compounds.

Polymers	Effective Factors	Bioactive Compounds	Main Achievements	Reference
Okra-gelatin	pH	Isoquercetin	Embedding inhibited the decomposition of isoquercetin caused by heat, acid, and enzymes.	[102]
Chitosan gelatin hydrogel	pH and Temperature	Antihypertensive drug ATL	Matrices fabricated by RSM design was found to be good device to release the ATL in a controlled pattern.	[103]
Polysaccharide hydrogel dressing	pH	Zn^2+^ and ferulic acid	Hydrogel has shown effectiveness in enhancing wound healing by influencing the immune microenvironment and facilitating the process of vascularization.	[104]
*Moringa oleifera* gum polysaccharides-poly (acrylic acid) hydrogel	pH	Ciprofloxacin	Non-Fickian diffusion mechanism. The hydrogels were observed to be pH responsive, non-haemolytic, mucoadhesive non-thrombogenic, and antioxidant in nature.	[105]
Gum ghatti hydrogel	pH	5-Fluorouracil	The release rate promoted as pH value rose.	[106]
Chitosan-oxidized Konjac glucomanan	pH	β-carotene	The structure may break down under neutral intestinal conditions (pH 7.0), resulting in the release of encapsulated antioxidants.	[107]
The glutamic acid grafted chitosan hydrogel beads	pH	Doxorubicin	Hydrogel beads showed pH-responsive, swelling and controlled release pattern.	[108]
Ethylenediaminetetraacetic acid-calcium-alginate	pH	Lactobacillus rhamnosus	The probiotic was successfully protected from the acidic environment of the stomach, allowing for a gradual release in the intestine.	[109]
Hemicellulose/graphene oxide (HC/GO) composite hydrogel (HGCH)	pH	B_12_	HGCH had high adaptability to soluble drugs and pH sensitivity triggered release.	[110]
Chitosan hydrogel	pH	DOX and bovine serum albumin	pH-responsive shrinkage enhanced the release of DOX 81% but prolonged BSA release.	[111]
Soy protein	Ionic strength	Lysozyme	When the ionic strength was raised to 0.5 M, a significant amount of lysozyme was released.	[112]
SA bio-nanocomposite magnetic hydrogel bead	pH	DOX	The hydrogel bead exhibited remarkable cytotoxicity to HT-29 cells.	[113]
Ovotransferrin–lysozyme	Ionic strength	Fatty acids	The enhancement of ionic strength through the electrostatic screening effect resulted in a reduction in complex stability and an increase in the release of free fatty acids.	[114]
Alginate	Solvent	Yerba mate extract	During release experiments, the maximum release of PC was observed in a 10% ethanolic solution.	[115]
Starch-chitosan films	Solvent	Polyphenols	The highest number of released polyphenols was observed in acidic medium.	[116]
Poly(curcumin-co-oxalate) copolymer	Redox	Curcumin	The copolymer’s dissociation in the presence of H_2_O_2_ resulted in a rapid release of the core.	[117]
Silica nanoparticles (MSN)–starch/maltodextrin/ maltose	Enzyme	Eugenol	Exogenous enzymes degrade the attached saccharides, leading to the release of eugenol and effective suppression of fungal growth.	[118]
Peptide-Peptide Hydrogels	Enzyme	Zidovudine	The release of the drug occurred through the hydrolysis resulting in the release of the drug in its unaltered state and subsequently diminishing the initial drug burst.	[119]
Phenoxy-alkyl maleate	Enzyme	Curcumin	The hydrogel structure underwent hydrolysis in an acidic environment or through the action of lysozyme, resulting in the liberation of the bioactive compound.	[120]
Maltodextrin (MD), inulin (IN), and oligofructose (OL)	Physical factors Temperature	Camu-camu (*Myrciaria dubia*) extract	Raising the temperature from 25 °C to 35 °C affected the release behavior of the compounds.	[121]
Poly (ethylene glycol)	Temperature	Tannic acid	The release rate of TA was elevated by higher bilayer numbers, increased pH, and elevated temperature, whereas it declined with rising ionic strength.	[122]
Succinylated cellulose monocrystal (Su-CNC) hydrogel	Temperature	Famotidine	The matrices exhibited a significant reaction to temperature variations, as evidenced by a reduction in their swelling behaviour and hydrophilicity at temperatures of 35 °C and above.	[123]
Copolymeric hydrogels (CH)	Temperature-Reduction-pH	5-fluorouracil	Temperature-, reduction-, and pH-responsive delivery of 5-fluorouracil from the CH system has been achieved.	[124]
Gelatine microsphere	Temperature	-	The diameter of microsphere increases with the increasing temperature.	[125]
Chitosan-poly dimethyl siloxane	Irradiation; Gamma	Curcumin	In a fibrous chitosan–curcumin polymer, exposure to 1 Gy gamma irradiation (^137^Cs) resulted in the release of 5 ± 1% of the conjugated curcumin, whereas 6 Gy irradiation released 98 ± 1%.	[126]
Hyaluronic acid-coumarin hydrogel	Near-infrared (NIR)	DOX	This injectable and biocompatible hydrogel may serve as an effective carrier for drug delivery responsive to near-infrared light, as well as for applications in bio-imaging.	[127]

**Table 3:** Chemical crosslinkers used in biopolymeric matrices for controlled delivery systems.

Crosslinkers	Biopolymers	Bioactive Ingredients	Main Achievements	Reference
Transglutaminase enzyme	Gelatin and chitosan Core–shell nanoparticles	Curcumin	Core–shell nanoparticles were found effective delivery vehicle for curcumin.	[175]
	Apricot kernel protein gels	Riboflavin	Riboflavin was protected from digestive degradation	[176]
	Gelatin film	Riboflavin and propranolol	Controlled release has been effectively demonstrated	[177]
	Casein/KGM hydrogels	Docetaxel	The hydrogel containing 1% KGM showed good drug release characteristics.	[178]
	Gelatin-based emulsion gels	β-carotene	Promising strategy for gelatin-emulsion gels with better stability, controlled release, and enhanced bioactive delivery system.	[179]
	Sodium caseinate film	Lysozyme	Lysozyme release was almost retarded	[180]
	Soy peptide nanogels	Resveratrol	This work provides a theoretical framework and technical aspects for the fabrication of peptide-based delivery systems.	[181]
	Gelatin film	Natamycin	Natamycin release was delayed and thus prolonged inhibition of *Aspergillus ochraceus*, *Aspergillus niger*, and *Penicillium funlculosu*.	[182]
Glutaraldehyde (GTA)	zein nanofibers	Eugenol (EU)	Controlled release of EU from zein encapsulation was enhanced through GTA vapor crosslinking.	[183]
	Chitosan microcapsules	Extract of red ginger oleoresin	Glutaraldehyde strengthened the bonds and lessened diameter of microspheres.	[184]
	Chitosan microcapsules	Ibuprofen	Drug release exhibited a biphasic pattern.	[185]
	Gelatin capsules	urea	Hydrophobically modification of gelatin shells for improved controlled release of urea.	[186]
	Chitosan microcapsules	Lysozyme	Release of lysozyme was successfully controlled.	[187]
	Gelatin-nanoparticles	Doxorubicin	Presented the fabrication of Ge-NPs for slow drug release and the application of ANN model	[188]
	Silk fibroin films	Rhodamine	Release rate increased linearly with the β-sheet content in the crosslinked film.	[189]
	Chitosan microparticles (Film and hydrogel types)	Metronidazole	The release profiles from hydrogel and film had better pattern compared to those containing drug powders, and hydrogel had preferrable outcome.	[190]
	Agarose films	Insulin	Prolonged insulin release was recorded.	[191]
Genipin	Gelatin hydrogel	Lidocaine	The ICG@Lido/gGel expressed potential as photothermal-triggered release cargo for PNB (Peripheral nerve block (PNB)).	[192]
	Chitosan/squid ring teeth protein hydrogels (CH/SRTs)	Curcumin	CH/SRTs delayed release of curcumin in a simulated gastrointestinal fluid.	[193]
	chitosan nanoparticles	catechin and quercetin	Both catechin and quercetin showed sustainable release profiles.	[194]
	β-casein micelles	Naringenin	Release of naringenin was delayed upon genipin crosslinking.	[195]
	Jackfruit gum-based nanoparticles	Curcumin	Offered high entrapment efficiency, controlled releases of curcumin, good mucoadhesive behaviour and enhanced anticancer activity.	[196]
	Gelatin hydrogel	Ascorbic acid	Infusion coefficient of water was the rate determining factor in this delivery vehicle.	[197]
	Chitosan Hydrogel	Thymoquinone, gefitinib, and erlotinib	Genipin-crosslinking showed stable loading and releasing with all three drugs.	[198]
	Gelatin-sodium caseinate microcapsules	Vitamin C and E	Fickian diffusion after crosslinking with genipin	[199]
	Chitosan hydrogel	Diclofenac	Hydrogels provided strong association with negatively charged diclofenac, limiting the release.	[200]
	Sodium caseinate-chitosan oligosaccharide nanoparticles	β-carotene	The β-carotene loaded had an enhanced anti-inflammatory activity.	[201]
	Chitosan-gelatin hydrogel	Metformin	Immediate release, with good safety profile, achieved by optimal genipin concentration	[202]
	Whey protein isolate chitosan hydrogel	Curcumin	Hydrogel had great advantages in continuous release of curcumin.	[203]
	Nanostructured lipid carrier-based hydrogel	Quercetin	Sustainable drug release was observed	[204]
	Chitosan hydrogels	Curcumin	Genipin cross-linked gels exhibited better release profile compared to glutaraldehyde crosslinked gels.	[205]
	Chitosan hydrogel	Iron fertilizer	GE-CSG@Fe could lower soil pH to preserve the activity of ferrous ion.	[206]
	Bovine serum albumin gel	Vitamin B6	Network mesh size, regulated by crosslinking, influences diffusion kinetics.	[140]
	Gelatin microneedles	Insulin	A practical device designed for noninvasive, painless, and controlled insulin delivery.	[207]
	Chitosan/alginate nanoparticles	α–mangostin	Nanoparticles have been suggested as viable options for the oral administration of drugs targeting the colon.	[208]
	Gelatin/chitosan films	Methylene blue	At pH 8.0, release flux decreased markedly compared to pH 1.5, indicating a highly controllable release.	[209]
	Whey protein isolate-coated liposomes	Flaxseed oil	Coating and crosslinking improved the chemical stability of flaxseed oil and modified system properties.	[210]
	Soy *β*-conglycinin nanoparticles	Curcumin	This protein-based vehicle could successfully transport insoluble bioactive in the gastric conditions.	[211]
Calcium chloride	Carboxymethyl cellulose (CMC) hydrogel	-	CMC hydrogel was successfully produced with CaCl_2_ as a crosslinker.	[212]
	Nanocellulose fibres (CNFs)-low methoxy pectin (LMP)-sodium alginate (SA) hydrogel	Clindamycin hydrochloride (CM)	Hydrogels composed of CNFs/LMP/SA at 1:1:1 and 2:0.5:0.5 mass ratios were successfully developed as biocomposite CNFs-based systems.	[213]
	Sodium alginate and gelatin filament	-	Micro- and nanomechanical properties of matrices can be tuned by altering biopolymer and crosslinker concentrations.	[214]
	The pequi (*Caryocar brasiliense*) mesocarp film	-	Calcium chloride promoted a reduction in solubility and an enhanced elongation.	[215]
	Alginate coating	Applied on fruit	Decreased respiratory rate & weight loss	[216]
	Chitosan and alginate hydrogel	Fertilizer (urea)	The hydrogel regulated urea release and encapsulation efficiency without altering its swelling in water.	[217]
	Alginate sodium beads	Valsartan	Beads followed the Korsmeyer–Peppas release mechanism, and finally showed extended-release beads	[218]
Glyoxal	Magnetic chitosan microspheres (GMS)	Doxorubicin (DOX)	It conferred GMS a potential candidate for DOX delivery.	[219]
	Chitosan hydrogel	Tannic acid	Tannic acid release from hydrogels was achieved, with its presence enhancing thermal stability.	[220]
	PVA/gelatin/guar gum hydrogels containing copper nanoparticles were developed as composites.	-	Hydrogels showed good blood compatibility, antibacterial, pH, and thermo-sensitive swelling behaviour.	[221]
Citric acid (CA)	Carboxymethylcellulose (CMCNa) and hydroxyethylcellulose (HEC) hydrogel	-	The findings revealed CA’s successful application as a crosslinking agent for the first time.	[222]
	Cellulose-based hydrogel	-	Citric acid increased the structural stability of the hydrogel.	[223]
	Nanocellulose fibres (CNFs)-low methoxy pectin (LMP)-sodium alginate (SA) hydrogel	Clindamycin hydrochloride (CM)	Hydrogels composed of CNFs/LMP/SA at 1:1:1 and 2:0.5:0.5 mass ratios were successfully developed as biocomposite CNFs-based systems.	[213]
	The pequi (*Caryocar brasiliense*) mesocarp film	-	Citric acid proved to be the optimal modifier for pequi mesocarp film properties.	[215]
Vanillin	Chitosan hydrogel		Crosslinking was markedly increased under microwave irradiation.	[224]
Multiple crosslinking agents				
Citric acid (CA) and Calcium chloride	Nanocellulose fibers (CNFs)-low methoxy pectin (LMP)-sodium alginate (SA) hydrogel	Clindamycin hydrochloride (CM)	Hydrogels composed of CNFs, LMP, and SA in 1:1:1 and 2:0.5:0.5 ratios were successfully developed as biocomposite systems.	[213]
	The pequi (*Caryocar brasiliense*) mesocarp film	-	Citric acid was found to be the best agent to modify the properties of pequi mesocarp films. Calcium chloride promoted a reduction in solubility and an enhanced elongation.	[215]
Tripolyphosphate and dextran sulfate	Chitosan-based core	Vitamin B_3_	Controlled drug release in a pH-independent manner.	[225]
Epichlorohydrin, calcium chloride and tannic acid	Chitosan/sodium alginate /tannic acid (CST) composite hydrogels	-	CST-31 made of a mass ratio of chitosan sodium alginate at 3:1 found to have suitable mechanical behavior, high porosity, water holding capacity, good biocompatibility and antibacterial activity.	[226]
Transglutaminase (TGase) and tannic acid (TA)	Gelatin-chitosan microcapsules	Lemon essential oil (LEO)	The dual cross-linked exhibited excellent antibacterial and antioxidant characteristics.	[227]
Transglutaminase (TGase) and ultrasound (US)	Lesser mealworm protein bead gel (LMPG)	B_12_	A high controlled release rate of B_12_ was achieved	[228]
Genipin/Zn^2+^.	*Spirulina* protein isolate (SPI) hydrogel	B_6_	Release profile showed the best fit to the Peppas–Sahlin model	[229]
Genipin and disulfide	Chitosan Hydrogel	Thymoquinone, gefitinib, and erlotinib	Genipin-crosslinking showed stable loading and releasing with all three drugs.	[198]

**Table 4:** Physical crosslinkers employed in controlled delivery systems.

Physical Crosslinkers	Biopolymers	Bioactive Ingredients	Main Achievements	Reference
Gamma ray	Chitosan and gelatin film	Quercetin	Irradiation prolonged the release and accelerated the retention of quercetin.	[239]
	Polyvinyl alcohol -polyethylene glycol hydrogels	Sodium sulphate	The release was a non-Fickian diffusion type	[240]
	Chitosan and gelatin film	Ferulic acid and tyrosol	Ferulic acid along with irradiation induced higher retention and lower diffusion.	[241]
	*Moringa oleifera* gum polysaccharides-poly (acrylic acid) hydrogel	Ciprofloxacin	Release occurred via a non-Fickian mechanism, and the hydrogels were pH-responsive, mucoadhesive, non-haemolytic, non-thrombogenic, and antioxidant.	[105]
	The glutamic acid grafted chitosan hydrogel beads	Doxorubicin	Hydrogel beads showed pH-responsive, swelling and controlled release pattern.	[108]
	Chitosan-polyvinyl alcohol coated polylactic acid bilayer film	Catechin	Gamma irradiation at 60 kGy decreased catechin release in high-fat simulant.	[153]
	PPy/PVP hydrogel	-	0.15PPy/PVP20 showed elevated compressive strength, reaching 51.96 ± 6.12 kPa.	[242]
	Fish gelatin film	Bamboo leave extract	Mechanical properties of matrices were enhanced with electron beam. Electron beam lessened the release rates.	[243]
Ultrasound (US)	Lesser mealworm protein bead gel (LMPG)	B_12_	A high controlled release rate of B_12_ was achieved	[228]
	Soy protein isolate (SPI) nanoparticles	Quercetin	Quercetin’s bio accessibility greatly improved by ultrasound assisted encapsulation of SPI.	[244]
Near-infrared (NIR)	Hyaluronic acid-coumarin hydrogel	DOX	This injectable and biocompatible hydrogel may serve as an effective carrier for drug delivery responsive to near-infrared light, as well as for applications in bio-imaging.	[127]
Ultraviolet ray	Chitosan hydrogel	DOX and bovine serum albumin	pH-responsive shrinkage enhanced the release of DOX 81% but prolonged BSA release.	[111]
	Aliphatic poly-globalide nanofibers	Rhodamine B and indomethacin	Cross-linking retained fibre morphology during swelling.	[245]
	Gelatin electrospun fibers	-	L929 cells exhibited superior growth on the UV-crosslinked scaffolds in comparison to those crosslinked with glutaraldehyde.	[246]
	Collagen-based nanofibers	5-Fluorouracil	UV-crosslinking prolonged the drug-release time from the fibers.	[247]
Microwave	Gelatin microspheres	-	A brief microwave exposure increased the swelling ratio to approximately 25%.	[125]
	Salecan-g-poly (*N*,*N*-dimethylaminoethyl acrylate) (PDMAEA) and nutgall tannic acid hydrogel	β-lactoglobulin (βlg)	βlg successfully accomplished both effective entrapment within the hydrogels and a release mechanism that is tunable and controlled by pH levels.	[248]
	Gum ghatti hydrogel	5-Fluorouracil	The release rate increased as the mesh size and pH levels rose.	[106]
	Chitosan and gelatin hydrogel	Antihypertensive drug ATL.	Matrices fabricated by RSM design was found to be good device to release the ATL in a controlled pattern.	[103]
	Chitosan hydrogel	-	The microwave irradiation technique can serve as a swift, dependable, and cost-effective approach for the cross-linking of chitosan.	[224]
	Carrageenan-guar gum hydrogel	Metronidazole	Developed IPN could be targeted controlled drug delivery cargo, allowing for expected swelling and drug release.	[97]
Heat Treatment	Polyelectrolyte multilayers on magnesium alloys	Gentamicin sulphate (GS)	This study demonstrated a better performance in antibacterial activities against *S. aureus* with a delayed release pattern of GS.	[249]
	Nanofibers of polyvinyl alcohol (PVA)	Curcumin	Heat treatment limited the release to a maximum of 20%, while UV exposure restricted it to 9%, in comparison with the non-crosslinked samples.	[250]
	Whey protein electrospun nanofibers	Rhodamine B	Heat-treated mat had slightly slower release rate.	[251]

**Table 5:** Plasticizers used in biopolymeric matrices.

Plasticizers	Biopolymers	Results Achieved	Reference
Polyethylene glycol (PEG) and glycerol	Sodium alginate (SA) and gelatin hydrogel.	PEG creates a plasticizing layer at the interface between sodium alginate and gelatin, whereas glycerol modifies the overall structure of the polymer matrix.	[253]
Glycerol	Sodium alginate/gelatin hydrogel	Demonstrates that the creation of new materials might not always be essential, as significant variations can arise from the same polymeric matrix across various aspects.	[254]
	Starch hydrogel	The degree of swelling was profoundly decreased in the presence of plasticizer.	[255]
	Ulvan hydrogel films	The swelling behaviour of the films were excellent and could be utilized as a wound dressing biopolymer.	[256]
Sorbitol	K-carrageenan and konjac glucomannan-based hydrogel film	40% (*w*/*w*) sorbitol-containing film demonstrated the most enhanced tensile strength.	[257]
Glycerol/sorbitol/water	Chitosan/sodium alginate films	Flexible films with suitable properties can be achieved through the utilization of either glycerol/water or sorbitol/water as combined plasticizers.	[258]
Glycerin	sodium alginate/poly(vinyl alcohol) (SA/PVA) hydrogel	The research indicates a notable reduction in the gel fraction, decreasing from 80.5 ± 2.1% to 45.0 ± 1.2% as the glycerin content is increased.	[259]
Ethylene glycol, glycerol, erythritol, xylitol, and sorbitol	Polyvinyl alcohol film (PVA)	Among the polyols tested, PVA plasticized with glycerol exhibited the greatest reduction in Young’s modulus, enhanced toughness, and the lowest melting temperature of 195 °C.	[260]
Essential oils (clove oil, oregano oil and tea tree oil)	Polyvinyl alcohol (PVA)-starch hydrogel	The hydrogel demonstrated excellent antibacterial, mechanical, and physical characteristics, making it suitable for use in wound dressing applications.	[261]
Glycerol (20–40%) and sorbitol (30–50%	Alginate	While glycerol demonstrates greater effectiveness as a plasticizer in terms of mass content, sorbitol exhibits superior plasticizing efficiency on a molecular level.	[262]
Glycerol, rapeseed oil, coconut oil, hazelnut oil, and sugars in the apple puree	Alginate and apple puree	The incorporation of vegetable oils and apple puree lowered the glass transition temperature (Tg), indicating their role as plasticizers.	[263]
Citric acid (CA)	Alginate	At high concentrations, CA exhibited a plasticizing effect.	[264]

## Data Availability

No new data were created or analyzed in this study.
